# A glibenclamide-sensitive TRPM4-mediated component of CA1 excitatory postsynaptic potentials appears in experimental autoimmune encephalomyelitis

**DOI:** 10.1038/s41598-022-09875-6

**Published:** 2022-04-09

**Authors:** Brenna C. Fearey, Lars Binkle, Daniel Mensching, Christian Schulze, Christian Lohr, Manuel A. Friese, Thomas G. Oertner, Christine E. Gee

**Affiliations:** 1 Institute of Synaptic Physiology, ZMNH, University Medical Center Hamburg-Eppendorf, Falkenried, 94 20251 Hamburg, Germany; 2 Institute of Neuroimmunology and Multiple Sclerosis, ZMNH, University Medical Center Hamburg-Eppendorf, Falkenried, 94 20251 Hamburg, Germany; 3 Division of Neurophysiology, University of Hamburg, 20146 Hamburg, Germany; 4 Present Address: Department of Psychological and Brain Sciences, Boston University, Boston, USA

**Keywords:** Multiple sclerosis, Ion channels in the nervous system, Neuroscience, Synaptic transmission

## Abstract

The transient receptor potential melastatin 4 (TRPM4) channel contributes to disease severity in the murine experimental autoimmune encephalomyelitis (EAE) model of multiple sclerosis and to neuronal cell death in models of excitotoxicity and traumatic brain injury. As TRPM4 is activated by intracellular calcium and conducts monovalent cations, we hypothesized that TRPM4 may contribute to and boost excitatory synaptic transmission in CA1 pyramidal neurons of the hippocampus. Using single-spine calcium imaging and electrophysiology, we found no effect of the TRPM4 antagonists 9-phenanthrol and glibenclamide on synaptic transmission in hippocampal slices from healthy mice. In contrast, glibenclamide but not 9-phenanthrol reduced excitatory synaptic potentials in slices from EAE mice, an effect that was absent in slices from EAE mice lacking TRPM4. We conclude that TRPM4 plays little role in basal hippocampal synaptic transmission, but a glibenclamide-sensitive TRPM4-mediated contribution to excitatory postsynaptic responses is upregulated at the acute phase of EAE.

## Introduction

In this study we tested the hypothesis that transient receptor potential melastatin 4 (TRPM4) channels are activated during, and contribute to, excitatory synaptic responses in CA1 hippocampal neurons. As we previously demonstrated that TRPM4-lacking mice have improved outcomes in the EAE model of multiple sclerosis and that *Trpm4*^*–/–*^ neurons are protected from excitotoxicity, we also examined the role of TRPM4 in synaptic transmission at the acute phase of EAE^1^.

TRPM4 are non-selective monovalent cation channels activated by intracellular calcium and modulated by ATP, calmodulin, protein kinase-c (PKC), phosphatidylinositol 4,5-bisphosphate, and H_2_O_2_^2–7^. It has been suggested that the TRPM4 channel mediates the Ca^2+^-activated non-selective cation current (*I*_CAN_) in a number of cell types^8^. TRPM4 channels are ubiquitously expressed in both excitable and non-excitable cells where they contribute to heart sinus rhythm^9,10^, boost smooth muscle cell depolarization^11^, and regulate calcium by decreasing the driving force in T cells and dendritic cells^12^. In the brain, TRPM4 antagonists reduce bursting in neurons of the substantia nigra, layer V of the medial entorhinal cortex and the thalamic reticular nucleus^13–15^. Similarly, in the pre-Bötzinger complex, TRPM4 contributes to bursting and inspiratory drive but not breathing rhythm generation^16–18^. In the hippocampus, knock-out of TRPM4 impairs theta-burst induced long-term potentiation (LTP) of synaptic transmission^19^ and in cerebellar Purkinje cells, both TRPM4 and TRPM5 contribute to, but are not absolutely required for the depolarization-induced slow current^20^. Recently, CA1 pyramidal neurons have been reported to express TRPM4 along the apical dendrites, accompanied by a TRPM4-dependent inward current^21^. TRPM4 may therefore be synaptically activated in CA1 neurons by calcium entering downstream of NMDA and metabotropic glutamate receptors. The additional depolarization due to TRPM4 could then further increase synaptically induced calcium entry. Such a positive-feedback process could play a physiological role, but if left unchecked may contribute to excitotoxicity.

Indeed, TRPM4 expression is altered in spinal neurons, glial cells, and the neuro-vasculature in response to injury^22–24^. In animal models of spinal cord injury and brain hemorrhage^25–28^, pharmacological blockade or knockout of TRPM4 improves clinical outcomes and also protects neurons against glutamate-induced excitotoxicity^1,29^. A recent study found that NMDA receptors and TRPM4 directly couple and disruption of this interaction is protective against excitotoxicity and in a mouse model of stroke^28^. Additionally, TRPM4 reportedly co-assembles with the sulfonylurea receptor 1 (SUR1)^30^. SUR1 is a member of the ATP-binding cassette transporter family and also co-assembles with the Kir6.2 inwardly-rectifying ATP-sensitive K^+^ channel^31^. In spinal cord injury, SUR1 but not Kir6.2 is upregulated^32^. SUR1 and TRPM4 upregulation have been reported in brain injury and in murine EAE^27,33,34^. The anti-diabetic drug, glibenclamide, antagonizes SUR1/Kir6.2, SUR1/TRPM4 and TRPM4 channels^35^ and the neuroprotective effects of glibenclamide treatment are similar to TRPM4 or SUR1 knock-out in EAE and brain injury models^1,33,36,37^.

In the hippocampus, the contribution of TRPM4 to basal synaptic transmission at the CA3-CA1 Schaffer collateral synapse remains poorly understood. Given that NMDAR-dependent calcium influx could activate TRPM4 and drive further depolarization and calcium entry, we measured CA3-CA1 excitatory postsynaptic calcium transients (EPSCaTs) in wild-type mouse organotypic hippocampal slice cultures and excitatory postsynaptic potentials (EPSPs) in acute hippocampal slices from wild-type and *Trpm4*^*–/–*^ healthy and EAE mice. We found no evidence for the involvement of TRPM4 in basal synaptic transmission. In EAE, we observed a reduction in EPSPs in the presence of glibenclamide in wild-type but not *Trpm4*^*–/–*^ or healthy mice suggesting a glibenclamide-sensitive TRPM4-mediated contribution to EPSPs during CNS inflammation.

## Results

### TRPM4 antagonism does not reduce calcium influx at individual CA1 pyramidal neuron spines or their parent dendrites

**Figure 1:**
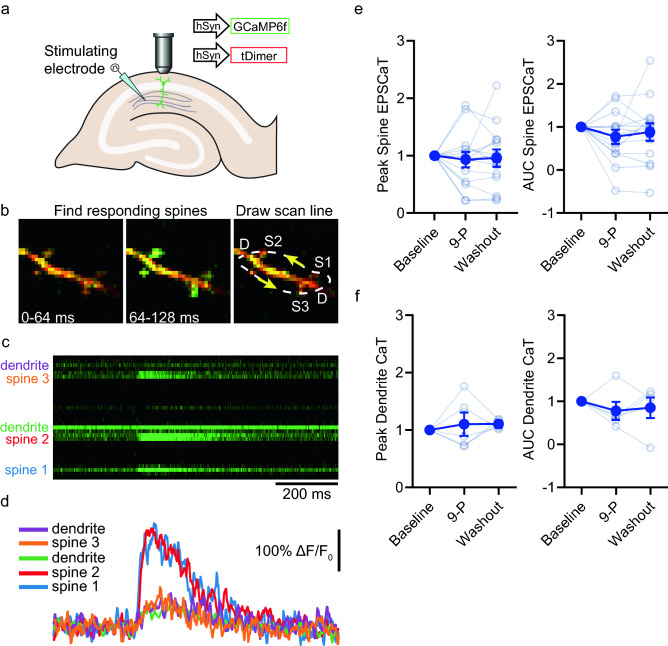
9-phenanthrol does not reduce evoked excitatory postsynaptic calcium transients. (**a**) A monopolar stimulating electrode was placed in stratum radiatum to stimulate the Schaffer collateral axons and evoke EPSCaTs in spines and dendrites of CA1 neurons expressing GCaMP6f. and tdimer2. EPSCaTs were recorded using a two-photon microscope. (**b**) Example trial in frame scan mode of an EPSCaT from responding spines (64–128 ms) and the corresponding arbitrary scan line passing through spines 1–3 and twice through the dendrite. (**c**) Example EPSCaTs in response to Schaffer collateral stimulation from the line in B. (**d**) Calcium transients of the individual spines from the linescan in C. (**e**) Baseline normalized spine EPSCaT peak and area under the curve (AUC) from 5 min. at baseline, during wash-in of 9-phenanthrol (9-P, 30 µM, last 5 min.) and following washout (n = 14 spines, individual responses, mean ± SEM). (**f**) Baseline normalized peak and AUC of dendritic calcium transients (CaTs). Individual dendritic responses are the average from two linescan crossings (n = 5 dendrites, individual responses, mean ± SEM).

To study the role of TRPM4 and calcium influx at the spine and dendrite, mouse organotypic hippocampal slice cultures were biolistically transfected with tdimer2 as a morphology marker and GCaMP6f. to monitor intracellular calcium of CA1 pyramidal neurons (Fig. 1a). Excitatory postsynaptic calcium transients (EPSCaTs) at single spines and dendrites in response to electrical stimulation of the Schaffer collaterals were monitored using a two-photon microscope (Fig. 1b). With the expectation that TRPM4 activation requires and contributes to large calcium transients, we aimed to induce not only isolated transients at single spines but responses at the dendrite as well (Fig. 1b–d). Washing in the TRPM4 antagonist, 9-phenanthrol, did not significantly reduce spine EPSCaT peak amplitude (Fig. 1e; *F*_*(2, 26)*_ = 0.6477, *p* = 0.53, RM-ANOVA) or area under the curve (AUC) (Fig. 1e; *F*_*(2, 26)*_ = 0.5971, *p* = 0.56, RM-ANOVA). Nor was there any reduction in dendritic CaT peak amplitude (Fig. 1f; *F*_*(2, 8)*_ = 0.8978, p = 0.44, RM-ANOVA) or AUC (Fig. 1f; *F*_*(2, 8)*_ = 1.364, *p* = 0.3, RM-ANOVA). Thus, TRPM4 does not contribute to synaptically evoked calcium transients at Schaffer collateral synapses.

### TRPM4 antagonists do not reduce CA1 excitatory synaptic potentials in healthy wild-type or Trpm4-deficient mice

**Figure 2:**
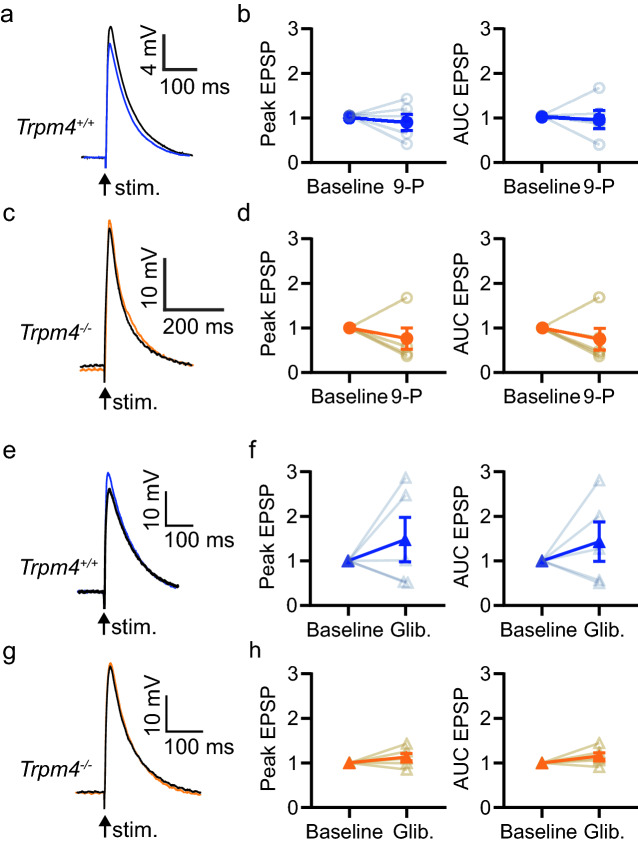
TRPM4 does not contribute to synaptic responses. (**a**–**d**) Effect of 9-phenanthrol (9-P, 30 µM) on EPSPs recorded from CA1 pyramidal neurons in acute slices from wildtype (*Trpm4*^+*/*+^) or knockout (*Trpm4*^*–/–*^) littermates. (**a**,**c**) Sample EPSPs before (blue/orange traces) or 20 min. after washing in 9-P (black). (**b**,**d**) Normalized peak and area under the curve (AUC) averaged from 5 min before (baseline) and the last 5 min in 9-P (n = 5, individual responses, mean ± SEM) (**e**–**h**) Effect of glibenclamide (Glib. 20 µM) on EPSPs recorded from CA1 pyramidal neurons in slices from wildtype (*Trpm4*^+*/*+^) or knockout (*Trpm4*^*–/–*^) littermates. (**e**,**g**) Sample EPSPs before (blue/orange traces) or 20 min. after washing in glibenclamide (black). (**f**,**h**) Normalized peak and area under the curve (AUC) averaged from 5 min before (baseline) and the last 5 min in glibenclamide (n = 6, individual responses, mean ± SEM). Non-normalized data and Cumming’s plots are shown in Supplemental Fig. 1.

We next prepared acute hippocampal slices from adult wild-type and *Trpm4*^*–/–*^ mice and made whole-cell patch-clamp recordings from CA1 pyramidal neurons. As action potentials, inhibitory potentials, and calcium-activated small conductance potassium (SK) channels might mask a TRPM4-mediated component of EPSPs, QX-314 was included in the intracellular solution to block voltage-gated Na^+^ channels, and bicuculline in the extracellular solution blocked GABA_A_ and SK channels^38^. D-serine was included in the extracellular solution to ensure that the glycine site of NMDA receptors was occupied. Large EPSPs (~ 40 mV deflections), expected to activate calcium channels, were recorded from CA1 pyramidal neurons in response to Schaffer collateral stimulation in *stratum radiatum*. There was no effect of 9-phenanthrol on EPSP amplitude or AUC in hippocampal slices from either wild-type (Fig. 2a,b, Supplemental Fig. 1; Wilcoxon paired test, amplitude *p* > 0.99; AUC *p* = 0.63) or *Trpm4*^*–/–*^ mice (Fig. 2c,d; Wilcoxon paired test, amplitude *p* = 0.1875; AUC *p* = 0.44). The TRPM4 antagonist glibenclamide also had no effect on the EPSP peak amplitude or AUC in either wild-type (Fig. 2e,f, Supplemental Fig. 2; Wilcoxon paired test amplitude *p* = 0.59, AUC *p* = 0.63) or *Trpm4*^*–/–*^ mice (Fig. 2g,h; Wilcoxon paired test amplitude *p* = 0.31; AUC *p* = 0.09). There were also no differences due to genotype in the cell parameters and baseline EPSP amplitude or AUC (Table 1). These results suggest that a TRPM4-mediated conductance does not significantly contribute to EPSPs.

**Table 1:** Only cell resistance significantly varies between genotype or health status at baseline.

Cell parameters at baseline	Healthy *Trpm4*^+*/*+^	Healthy *Trpm4*^*–/–*^	EAE *Trpm4*^+*/*+^ (*p* vs healthy *Trpm4*^+*/*+^)	EAE *Trpm4*^*–/–*^	2-way ANOVA genotype EAE
Number of cells	10	11	12	10	–
Peak amplitude (mV)	28.8 ± 7.6	38.6 ± 7.7	41.9 ± 9.1	41.5 ± 7.1	F = 0.196 *p* = *0.66* F = 0.7 *p* = *0.39*
Resting membrane potential (mV)	− 72.0 ± 4.8	− 78.3 ± 3.5	− 69.6 ± 1.8	− 70.5 ± 2.7	F = 1.097 *p* = *0.30* F = 3.066 *p* = *0.087*
Total resistance (MΩ)	221 ± 19	265 ± 12	279 ± 17 (*p* = 0.046*)	314 ± 19	F = 6.381, *p* = 0.0154* F = 11.54 *p* = 0.0015*
Area under the curve (mV*ms)	1945 ± 638	3110 ± 709	2966 ± 836	2700 ± 486	F = 0.368 *p* = *0.547* F = 0.15 *p* = *0.698*
Half-width (ms)	47.2 ± 4.3	60.8 ± 3.8	53.0 ± 7.3	55.0 ± 5.1	F = 2.996 *p* = *0.0908* F = 0.0596 *p* = *0.808*
Rise slope (mV/ms)	3.6 ± 0.71	2.85 ± 0.21	4.53 ± 1.56	3.46 ± 0.96	F = 0.8503 *p* = *3617* F = *0.649* *p* = *0.452*
Rise time (ms)	4.8 ± 0.62	7.57 ± 1.37	7.52 ± 1.23	8.47 ± 1.30	F = 2.370 *p* = *0.131* F = 2.240 *p* = *0.142*
Clinical score	N/A	N/A	3.08 ± 0.11 *n* = 6 mice	3.00 ± 0.26 *n* = 6 mice	t(10) = 0.2988 *p* = *0.77*
Body weight	N/A	N/A	19.88 ± 1.08 *n* = 6 mice	20.43 ± 1.24 *n* = 6 mice	t(10 ) = 0.3346 *p* = *0.74*

All measurements are taken from the baseline prior to drug wash-in. Data are mean ± SEM. The degrees of freedom are (1, 42) for all parameters except clinical score and body weight. Upper pair of F and p values are for genotype (*Trpm4*^+*/*+^ vs *Trpm4*^*–/–*^) comparisons and the lower pair are for treatment (EAE vs. healthy) comparisons. For clinical score and body weight, unpaired t-tests were used. *n*-size is indicated within each cell.

### Glibenclamide but not 9-phenanthrol reduces EPSP peak amplitude in wild-type EAE mice

**Figure 3:**
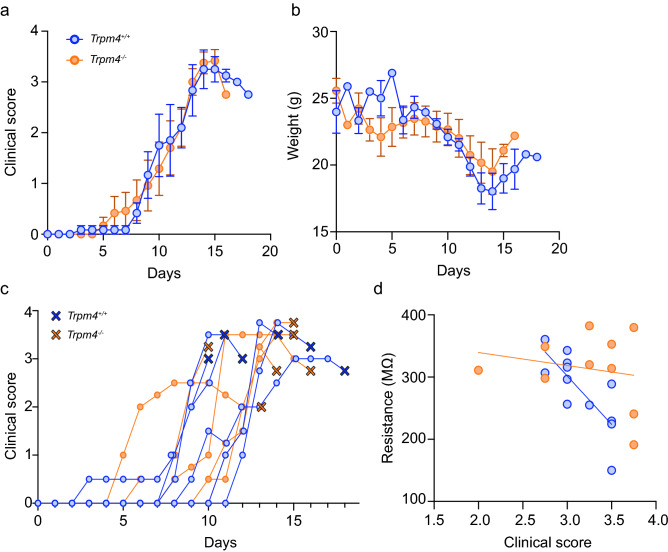
The induction and acute phase of EAE was not different in *Trpm4*^*–/–*^ and *Trpm4*^+*/*+^ littermates. (**a**) Daily clinical scores and (**b**) body weight of *Trpm4*^*–/–*^ (orange circles, n = 6 mice) and *Trpm4*^+*/*+^ (blue circles, n = 6 mice) littermates recorded daily after induction of experimental autoimmune encephalomyelitis (EAE) on day 0 (mean ± SEM). (**c**) EAE clinical progression of individual mice and score at time of sacrifice for electrophysiology (X’s). (**d**) Cell resistance was calculated from 200 ms–10 pA current injections in drug-free ACSF. Cell resistance is negatively correlated with the final clinical score in *Trpm4*^+*/*+^ mice (Pearson’s r = − 0.77, p = 0.0035) but not in *Trpm4*^*–/–*^ mice (Pearson’s r = − 0.2, p = 0.58).

Several studies have suggested that TRPM4 function plays an important role in diseased states, including models of multiple sclerosis^1,29^, ischemia^25,39^, and brain injury^40^. Therefore, we induced EAE in *Trpm4*^+*/*+^ and *Trpm4*^*–/–*^ littermates to test whether TRPM4-dependent currents only significantly contribute to synaptic transmission in an inflamed state. Clinical scores and body weight were measured daily (Fig. 3a,b). Acute slices were prepared at the peak of the acute phase, i.e. a clinical score of at least 3 or a decreasing score, blind to genotype (Fig. 3a). There were no differences due to genotype in the clinical score or body weight at the peak of acute EAE, as previously reported (Fig. 3a,b; Table 1)^1^.

EPSPs were recorded from EAE mice following the same procedure as for the healthy mice. Table 1 summarizes the baseline cell parameters, EPSP amplitude and AUC of CA1 neurons from healthy and EAE, *Trpm4*^+*/*+^ and *Trpm4*^*-/-*^ mice. There were no differences due to genotype or health in nearly all parameters (two-way ANOVA). Only cell resistance showed a significant difference due to genotype (Table 1; *F*_*(1, 42)*_ = 6.38, *p* = 0.015) and between healthy and EAE mice (Table 1; *F*_*(1, 42)*_ = 11.54, *p* = 0.0015) and a post-hoc Sidak’s test for multiple comparisons revealed a significant difference between healthy *Trpm4*^+*/*+^ and EAE *Trpm4*^+*/*+^ (Table 1; p = 0.046). Interestingly, in the EAE slices there was a significant negative correlation of cell resistance with clinical score in *Trpm4*^+*/*+^ neurons that was absent in *Trpm4*^*–/–*^ neurons (Fig. 3d; *Trpm4*^+*/*+^: *R*^*2*^ = 0.57, *p* = 0.004; *Trpm4*^*–/–*^: *R*^*2*^ = 0.05, *p* = 0.58).

**Figure 4:**
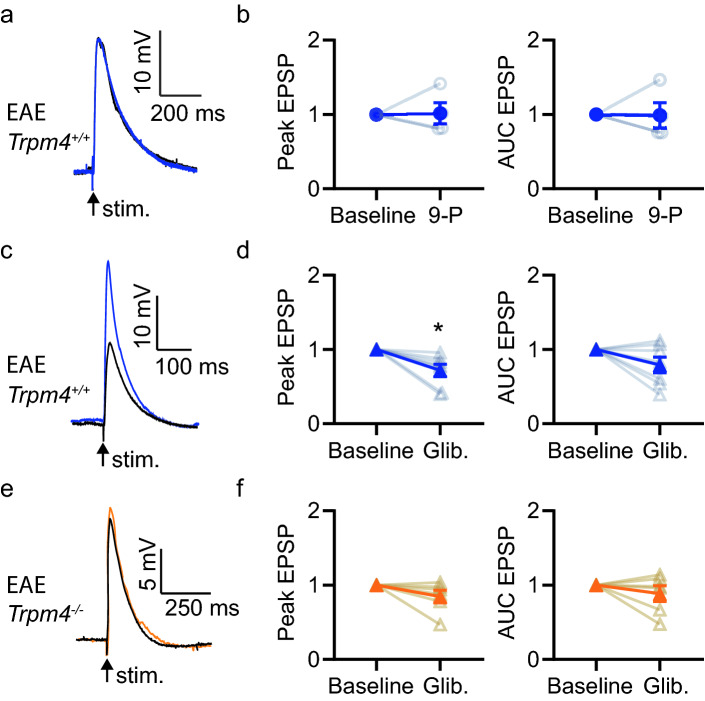
Glibenclamide, not 9-phenanthrol, reduces EPSP peak amplitude in CA1 pyramidal neurons in EAE mice. (**a**) EPSPs recorded from a representative CA1 pyramidal neuron in response to stimulation of stratum radiatum in an acute slice from a *Trpm4*^+*/*+^ mouse prepared during the acute response to EAE. After establishing a stable baseline (blue trace), 9-phenanthrol (30 μM, 9-P) was washed into the bath for 20 min (black trace). (**b**) Normalized average EPSP peak and area under the curve (AUC) at baseline (average 5 min) and after wash-in of 9-phenanthrol (average 15–20 min) (n = 4, individual experiments, mean ± SEM). (**c**) As in a but black trace is after wash-in of glibenclamide (20 µM, Glib). (**d**) As in B but before and after washing in glibenclamide (n = 8, individual experiments, mean ± SEM, *p = 0.02 Wilcoxon paired test). (**e**) As in C but example EPSPs were recorded before (orange trace) and after washing in glibenclamide (black trace) onto a CA1 neuron in a slice made from a *Trpm4*^*-/-*^ EAE littermate. (**f**) As in D but from *Trpm4*^*-/-*^ littermates (n = 7, individual experiments, mean ± SEM). Non-normalized data and Cumming’s plots are in Supplemental Fig. 2.

As in healthy animals, 9-phenanthrol had no apparent effect on EPSP amplitude or AUC in slices made from EAE *Trpm4*^+*/*+^ mice (Fig. 4a,b, Supplemental Fig. 2; amplitude *p* = 0.63; AUC *p* = 0.25). Given there was no drug effect in *Trpm4*^+*/*+^ neurons, we had no need to test for off-target effects and elected not to treat slices from *Trpm4*^*–/–*^ animals with 9-phenanthrol. In contrast to 9-phenanthrol, glibenclamide significantly though modestly reduced the EPSP amplitude (Fig. 4c,d, Supplemental Fig. 2; Wilcoxon paired test *p* = 0.02) but not the AUC (*p* = 0.38) in *Trpm4*^+*/*+^ neurons. The reduction of EPSP amplitude by glibenclamide was absent in *Trpm4*^*–/–*^ mice (Fig. 4e,f, p = 0.16), indicating a TRPM4-specific effect. The AUC remained unchanged in CA1 neurons from both *Trpm4*^+*/*+^ and *Trpm4*^*–/–*^ mice (Fig. 4f; *Trpm4*^+*/*+^ *p* = 0.38; *Trpm4*^*–/–*^* p* = 0.56).

### TRPM4 mRNA levels do not change in EAE

**Figure 5:**
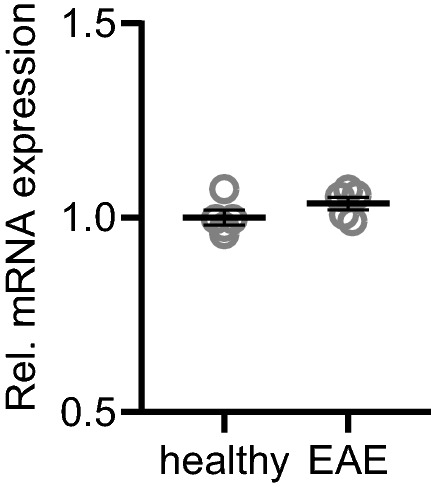
TRPM4 mRNA expression is unchanged in cortical neurons from healthy and EAE mice. Cortical nuclei from healthy and EAE-induced mice were sorted by NeuN immunoreactivity. TRPM4 expression levels were assessed by quantitative PCR. n = 5; unpaired 2-tailed t-test, p = 0.19.

As the recordings from EAE mice were suggestive of a change in TRPM4 function, localization or regulation during EAE, we were specifically interested in whether neuronal TRPM4 expression might be altered in EAE. We isolated cortical neuron nuclei from healthy and EAE mice and performed a quantitative polymerase chain reaction analysis to determine mRNA expression levels of TRPM4. Interestingly, TRPM4 mRNA levels did not change under EAE (Fig. 5, t-test, *p* = 0.19) .

Given no change in mRNA levels between healthy and EAE mice, we sought to determine whether we could detect differences in TRPM4 localization or protein levels. We first performed immunohistochemistry in hippocampal CA1 from healthy *Trpm4*^+*/*+^ *and Trpm4*^*-/-*^ mice.We used 3 commercially available antibodies and one antibody generously provided by Simard^24,35^. The Alomone antibody was raised in rabbits against a short peptide of amino acids (aa) 5–17 of human TRPM4 (intracellular N-terminus), the Abcam antibody was raised in rabbits against a synthetic peptide from the C-terminal region of mouse TRPM4, the Santa Cruz antibody was raised in goat against a peptide mapping within an internal region of human TRPM4, and the antibody from Dr. J. M. Simard’s lab was raised in rabbits against aa 1–612 of mouse TRPM4 (intracellular N-terminus). Disappointingly, none of the four antibodies showed differential staining patterns in hippocampal slices of *Trpm4*^+*/*+^ and *Trpm4*^*–/–*^ mice although it appeared there was clear staining around the soma and dendrites (Supplemental Fig. 3). Thus, we could not determine the expression pattern of TRPM4 protein in CNS tissue and did not attempt to stain tissue from EAE mice.

## Discussion

In this study, we found that TRPM4 does not participate in basal synaptic transmission at the CA3-CA1 Schaffer collateral synapse in organotypic hippocampal slice cultures or in acute hippocampal slices made from healthy mice. We did however, detect a small TRPM4-dependent effect at the acute phase of EAE.

We undertook this study expecting to find that TRPM4 indirectly boosted synaptic EPSCaTs and directly increased EPSPs in the hippocampus, particularly when synaptic stimulation was strong enough to cause dendritic calcium rises typical of NMDA spikes or large EPSPs^41^. We blocked postsynaptic action potentials, inhibitory GABA_A_ receptors and SK channels, which are also activated by calcium and hyperpolarize rather than depolarize spines and dendrites, and still did not see evidence of a TRPM4 conductance in slices from healthy mice. Surprisingly, the antagonist glibenclamide, but not 9-phenanthrol, decreased EPSP amplitude in EAE *Trpm4*^+*/*+^ mice. Importantly, glibenclamide did not affect EPSPs in EAE *Trpm4*^*–/–*^ mice. Glibenclamide is considered less specific to TRPM4 than 9-phenanthrol^42^ and glibenclamide is widely known for its ability to antagonize SUR1-Kir6.2 (i.e. K_ATP_) channels^43^. It has been shown that SUR1-TRPM4 channels also co-assemble and that glibenclamide has a 100-fold higher efficacy for SUR1-TRPM4 channels than for TRPM4 homomeric channels^35^. Interestingly, in hypoglycemic conditions glibenclamide decreases EPSCs in cortical neurogliaform cells but not pyramidal neurons via K_ATP_ channels^44^. While cortical and hippocampal pyramidal neurons should not be considered identical, they are similar morphologically and electrophysiologically and TRPM4 seems not to participate in excitatory synaptic transmission in both. We cannot rule out that K_ATP_ (SUR1-Kir6) channels are also down-regulated in the *Trpm4*^*–/–*^ mice although we think it more likely that glibenclamide in EAE reduces EPSPs via SUR1-TRPM4 or TRPM4 channels in the slices from EAE mice.

Although TRPM4 mRNA is present in neurons^1,28^ we were unable to detect TRPM4 protein in brain slices by immunostaining. The fluorescent signals seen with several antibodies was unspecific as it was the same in slices from *Trpm4*^*–/–*^ and *Trpm4*^+*/*+^ mice. It may be that protein expression of TRPM4 is simply too low in the hippocampus to be detected by immunohistochemistry. Alternatively, antigenic sites may be masked in tissue, or the antibodies recognize some endogenous protein that is not TRPM4 and is therefore present in both the wild-type and TRPM4-lacking mice. A recent study demonstrated somato-dendritic immunostaining against TRPM4 in CA1 neurons of the hippocampus, however, there was no published validation of these antibodies in *Trpm4*^*–/–*^ tissue^21^.

Our findings that TRPM4 plays little to no role in hippocampal synaptic transmission, support a recent study, describing a direct coupling of NMDA receptors with TRPM4^28^. When this coupling, which is suggested to underlie the role of TRPM4 in excitotoxicity, is disrupted there is no change in calcium influx or in basal synaptic transmission. Although TRPM4 antagonism is a different action than disruption of NMDA/TRPM4 interaction, in neither ours nor their study was there an apparent involvement of TRPM4 in normal synaptic transmission. The NMDA/TRPM4 coupling is hypothesized to occur specifically at extrasynaptic sites^28^, which are typically activated only under conditions of massive glutamate release such as during seizures or in neurodegeneration^45^. Possibly our findings reflect an increased synaptic NMDA/TRPM4 interaction that moderately boosts basal synaptic transmission under pathological conditions like that induced by EAE. The modest effect might be due to only subtle inflammatory changes in the hippocampus during EAE. Interestingly, we did not observe a change in the mRNA levels of TRPM4 in cortical pyramidal neurons between healthy and EAE animals. While this does not preclude an increase in TRPM4 protein in hippocampal pyramidal neurons, it suggests that a re-organization rather than upregulation may occur as we observed in spinal cord^1^.

In conclusion, we observed that TRPM4 does not significantly contribute to basal synaptic transmission at the Schaffer collateral synapse of healthy mice, but TRPM4 may contribute to EPSPs in EAE. In multiple sclerosis and EAE there are clear hippocampus-specific cognitive impairments, synaptic dysfunction and atrophy both in patients and in animal models^46,47^. TRPM4 might prove an interesting therapeutic target if it is found that cognitive function after i.e. EAE is improved by TRPM4 inhibitors as in chronic cerebral hypoperfusion^48^. The relatively small contribution to synaptic transmission may cause fewer side effects and prove advantageous over direct targeting of, for instance, NMDA receptors.

## Materials and methods

### Animals

All transgenic mice (Trpm4(tm1.1Mfre)^49^ and wild type C57BL/6 were housed and bred at the University Medical Center Hamburg-Eppendorf (UKE) animal facility. Both female and male mice were used as indicated. All mice were housed with a 12-h light/dark cycle and had water and food ad libitum. All procedures performed in mice were in compliance with German law and according to the guidelines of Directive 2010/63/EU. Protocols were approved by the local ethics committee (Behörde für Justiz und Verbraucherschutz (BJV), Lebensmittelsicherheit und Veterinärwesen). The ARRIVE guidelines for reporting the experiments were followed. No a priori determination of sample size was performed.

### Mouse organotypic hippocampal slice cultures

Hippocampal slice cultures were prepared from P4-P7 male wild type C57BL/6 mice following a published protocol^50^. Pups were anesthetized with 80% CO_2_/20% O_2_ and decapitated. Hippocampi were dissected in cold slice culture dissection medium containing (in mM): 248 sucrose, 26 NaHCO_3_, 10 glucose, 4 KCl, 5 MgCl_2_, 1 CaCl_2_, 2 kynurenic acid and 0.001% phenol red. The solution was saturated with 95% O_2_, 5% CO_2_, pH 7.4, 310–320 mOsm kg^−1^. Tissue was cut into 400 µM thick sections on a tissue chopper and cultured on porous membranes (Millipore PICMORG50) at 37 °C in 5% CO_2_. No antibiotics were added to the slice culture medium which was partially exchanged (60–70%) twice per week and contained (for 500 ml): 394 ml Minimal Essential Medium (Sigma M7278), 100 ml heat inactivated donor horse serum (H1138 Sigma), 1 mM L-glutamine (Gibco 25030–024), 0.01 mg ml^−1^ insulin (Sigma I6634), 1.45 ml 5 M NaCl (S5150 Sigma), 2 mM MgSO_4_ (Fluka 63126), 1.44 mM CaCl_2_ (Fluka 21114), 0.00125% ascorbic acid (Fluka 11140), 13 mM D-glucose (Fluka 49152).

### Plasmid construction and expression

DNA encoding GCaMP6f and tdimer2 were each subcloned into a neuron-specific expression vector (pCI) under the control of the human synapsin-1 promoter using the HindIII and NotI restriction sites for GCaMP6f and the Acc65I and EcoRI restriction sites for tdimer2. To express GCaMP6f and tdimer2 in CA1 pyramidal neurons, gold particles (1.6 μm, 2.75 μg DNA per mg gold) were coated with an 8:3 ratio of expression vectors encoding GCaMP6f and tdimer2 (respectively). At DIV 7–9, slice cultures were ballistically transfected using a Helios gene gun (Bio-Rad). Experiments were conducted between DIV 14–21.

### Two-photon microscopy

The two-photon imaging setup was custom built based on an Olympus BX51WI microscope. An Olympus LUMPlan W-IR2 60 × 0.9 NA objective was used and image acquisition was controlled by the open-source software package ScanImage 3.7^51^, which we modified to allow user-defined arbitrary line scans. A pulsed Ti:Sapphire laser (Chameleon, Coherent) was used to excite GCaMP6f and tdimer2 at 980 nm. Emitted photons were collected through the objective and oil-immersion condenser (1.4 NA, Olympus) with two pairs of photomultiplier tubes (H7422P-40, Hamamatsu). 560 DXCR dichroic mirrors and 525/50 and 607/70 emission filters (Chroma) were used to separate green and red fluorescence. Excitation light was blocked by short-pass filters (ET700SP-2P, Chroma).

### Calcium imaging

An organotypic hippocampal slice culture was transferred to the chamber at the two-photon microscope and bathed in oxygenated ACSF containing (in mM): 135 NaCl, 2.5 KCl, 4 CaCl_2_, 4 MgCl_2_, 10 Na-HEPES, 12.5 D-glucose, 1.25 NaH_2_PO_4_. To identify spines and dendrites responding to electrical stimulation of Schaffer collateral axons (two 0.2 ms paired pulses with an interpulse interval of 40 ms), frame scans (at 15.625 Hz) of oblique dendrites were acquired. After finding a responding spine, user-defined arbitrary line scans (500 Hz) that crossed the responding spines and dendrite were used to quantify the calcium response to stimulation, improving time resolution and signal to noise. Responses to stimulation were acquired at 0.0167 Hz. A stable baseline was acquired for at least twenty minutes. After baseline, 9-phenanthrol (30 μM) was washed into the chamber and responses were acquired for another twenty minutes. Finally, fresh ACSF containing no drugs was washed into the chamber and responses were acquired for a final twenty minutes. For analysis, regions of interest (ROI) were drawn over spines and dendrites in ImageJ and the relative percent change in GCaMP6f. green fluorescence was calculated as 100 * (F-F_0_) / F_0_ where F is the fluorescence intensity and F_0_ is the average fluorescence intensity prior to stimulation. Peak amplitude and area under the curve (AUC) were extracted from within the response time-window after smoothing in Matlab with a moving average span of 5. Baseline, drug and washout responses over each twenty minute period were then averaged (Fig. 1).

### Immunohistochemistry

*Trpm4*^+*/*+^ and *Trpm4*^-/-^ mice were anaesthetized with an intraperitoneal injection of 10 mg ml^−1^ esketamine hydrochloride (Pfizer), 1.6 mg ml^−1^ xylazine hydrochloride (Bayer) in water (100 µl per 10 g of body weight). Mice were then perfused with 0.1 M phosphate buffer and fixed in 4% paraformaldehyde (PFA). For immunohistochemistry, 40 µm thick free floating sections were washed 3 × 5 min. in 1 × PBS followed by 10 min. in 0.5% sodium borohydride (Sigma #S-9125) in 1 × PBS. Slices were again washed 3 × 5 min. in 1 × PBS. Slices were incubated in blocking solution for 1 h (0.3% BSA Sigma #A-4503, 10% horse serum Invitrogen #16,050–130, 0.3% Triton X-100). Slices were then incubated overnight at 4 °C in Carrier + (0.2% BSA, 1% horse serum and 0.3% Triton X-100) with primary antibody against TRPM4 (Rabbit polyclonal, 1:200, Alomone ACC-044; rabbit polyclonal, 1:100, Abcam ab104572; goat polyclonal, 1:500, Santa-cruz SC-27540). TRPM4 rabbit anti-serum (targeting the N-terminus), a gift from Dr. J Marc Simard (University of Maryland School of Medicine), was reconstituted in water and used at a concentration of 1:200. The next day slices were washed 3 × 5 min. In 1 × PBS. Secondary goat anti-rabbit antibodies coupled to Alexa-488 (Invitrogen Cat. # A-11008, 1:1000) in Carrier + were incubated with slices for 2–5 h. Slices were then mounted and imaged with a confocal microscope (Leica) for Abcam and Dr. Simard’s antibodies with a 63 × oil immersion objective [HCX PLA PO 63 × NA 1.32] and Olympus Fluoview FV 1000 with a 60 × oil immersion objective [UPLSAPO 60 × NA 1.35] for Alomone and secondary only). Excitation/emission spectra and filters for Alexa-488 were selected using the automatic dye selection function of the confocal software. Acquisitions were taken with the same laser intensity and PMT voltages for samples acquired within the same batch of stainings. The antibodies from Alomone and Dr. Simard target the N-terminus and the Abcam antibody targets the C-terminus of TRPM4.

### Experimental autoimmune encephalomyelitis (EAE) induction

Male and female *Trpm4*^-/-^ and their wild-type (*Trpm4*^+*/*+^) littermates were used for EAE (Trpm4(tm1.1Mfre)^49^) induction at 3–5 months old. We immunized mice subcutaneously with 200 μg MOG_35–55_ (EP02030, peptides&elephants, Hennigsdorf, Germany) in complete Freund's adjuvant (BD) containing 2 mg ml^−1^ *Mycobacterium tuberculosis* (H37Ra, BD). We injected 200 ng pertussis toxin (EMD Millipore) intraperitoneal on the day of immunization and 48 h later. We scored the mice daily for clinical signs by the following system: 0, no clinical deficits; 1, tail weakness; 2, hind limb paresis; 3, partial hind limb paralysis; 3.5, full hind limb paralysis; 4, full hind limb paralysis and forelimb paresis; 5, premorbid or dead. Hippocampal slices were prepared at scores ≥ 3 or shortly after the score began to decrease 11–18 days after EAE induction (see Electrophysiology, below).

### Expression analysis of Trpm4 in cortical neurons from healthy and EAE mice

Healthy and EAE mice in the chronic phase of the disease were sacrificed with CO_2_. Cortices were homogenized in EZ buffer (Sigma) and the 500 g pellet washed once. Nucleic pellets were resuspended in Nuclei Buffer (NB, 430 mM sucrose, 2 mM MgCl_2_, 25 mM KCl, 65 mM glycerophosphate, 5% glycerol, 1 mM EDTA, 1% BSA) and washed twice. Final pellets were resuspended in NB + 0.2U/µl Ribolock (Thermofisher) and passed through MACS filters. Nuclei were stained using anti-NeuN-AF647 (1:1000) and PI (1:2000) and NeuN positive nuclei were sorted into 5 ml tubes. Total RNA was extracted with RNeasy Mini Kit (Qiagen) and mRNA reverse transcribed to obtain cDNA for quantitative PCRs.

### Electrophysiology

Female and Male *Trpm4*^+*/*+^ and *Trpm4*^-/-^ mice were sacrificed between 2 and 6 months of age. Mice were briefly anesthetized with 80% CO_2_ /20% O_2_ prior to decapitation. The brain was dissected and immersed in ice-cold solution containing (in mM): 110 choline chloride, 25 NaHCO_3_, 25 D-glucose, 11.6 sodium L-ascorbate, 7 MgSO_4_, 1.25 NaH_2_PO_4_, 2.5 KCl, 0.5 CaCl_2_, continuously bubbled with 95% O_2_ and 5% CO_2_, pH 7.4. Coronal slices (300 μm thick) were cut using a vibratome and were allowed to recover at 34 °C for 30 min in oxygenated artificial cerebrospinal fluid (ACSF) containing (in mM): 125 NaCl, 26.2 NaHCO_3_, 11 D-glucose, 1 NaH_2_PO_4_, 2.5 KCl, 1.3 MgCl_2_, 2.5 CaCl_2_. Slices were then kept in the same solution at room temperature until used. After at least 1 h at room-temperature, a slice was placed in the recording chamber and continuously perfused with oxygenated ACSF supplemented with D-Serine (30 μM) to occupy the second agonist binding site of NMDA receptors, bicuculline-methochloride (10 μM) to block GABA_A_ receptors and SK-channels^38^ and 0.1% DMSO (see below). Recordings were performed using either a Multiclamp 700B or an Axopatch 200B amplifier (Molecular Devices). Recordings were controlled and digitized using National Instruments A/D boards and Ephus software in the Matlab environment^52^. Current-clamp recordings from CA1 pyramidal cells were performed at 23–25 °C (Figs. 2, 3) or at 30–32 °C (Fig. 4). Patch pipettes with a tip resistance of 3–5 MΩ were filled with (in mM): 135 K-gluconate, 10 HEPES, 4 MgCl_2_, 4 Na_2_-ATP, 0.4 Na-GTP, 10 Na_2_-phosphocreatine, 3 L-ascorbic acid and 3 QX-314 chloride (to block voltage-gated sodium channels). For recordings done in healthy *Trpm4*^+*/*+^ and *Trpm4*^*-/-*^ mice, the experimenter was not blind to genotype. Recordings made from EAE mice were performed blind to the genotype. To evoke excitatory postsynaptic potentials (EPSPs), a monopolar electrode was placed in the *stratum radiatum* and 0.2 ms pulses were delivered using an ISO-Flex stimulator (A.M.P.I.). The stimulation intensity was set to produce an EPSP of approximately 40 mV. After a stable baseline was achieved (at least five minutes), a TRPM4 antagonist, either 9-phenanthrol (30 μM) or glibenclamide (20 μM), was washed into the bath. Both 9-phenanthrol and glibenclamide were dissolved in DMSO and resulted in a final 0.1% DMSO total concentration in the ACSF. An equivalent concentration of DMSO was included in the drug-free ACSF. Trials were acquired at 0.08–0.1 Hz.

### Analysis & statistics

Calcium imaging experiments were analyzed using ImageJ and repeated-measures ANOVA was used for statistical analysis. Electrophysiology analysis was completed in Matlab. Individual trials were then averaged into one minute bins. For statistics, averages were made from the last five minutes of baseline and within 15–22 min after washing in the drug. Analysis of electrophysiological recordings from EAE animals was conducted blind. All statistics were performed using Graphpad Prism (version 8). For paired analyses, the Wilcoxon matched pairs signed rank test was used. For analysis of the role of genotype and clinical score, a two-way ANOVA was used for each parameter. *p* less than 0.05 was considered significant. 5000 bootstrap samples were taken; the confidence interval is bias-corrected and accelerated. To calculate estimation statistics, multiple paired differences were calculated using the estimation statistics app^53^. The P-value(s) reported are the likelihood(s) of observing the effect size(s), if the null hypothesis of zero difference is true. For each permutation P-value, 5000 reshuffles of the control and test labels were performed.

## Supplementary Information

Supplementary Information 1.

Supplementary Information 2.
