# Supplementary material for: A glibenclamide-sensitive TRPM4-mediated component of CA1 excitatory postsynaptic potentials appears in experimental autoimmune encephalomyelitis
Source: Sci Rep. 2022 Apr 9;12:6000. doi: 10.1038/s41598-022-09875-6 (PMC8994783; doi:10.1038/s41598-022-09875-6)
Supplement: Supplementary file 2 — Supplementary Information 2. [file 41598_2022_9875_MOESM2_ESM.pdf]

**Supplemental Figures for:**

# A glibenclamide-sensitive TRPM4-mediated component of CA1 excitatory postsynaptic potentials appears in experimental autoimmune encephalomyelitis

Short Title: TRPM4 in EAE

Brenna C. Fearey[1,4], Lars Binkle[2], Daniel Mensching[2], Christian Schulze[1], Christian Lohr[3], Manuel A. Friese[2], Thomas G. Oertner[1], Christine E. Gee[1]*

[1] Institute of Synaptic Physiology, ZMNH, University Medical Center Hamburg-Eppendorf, Falkenried 94 20251 Hamburg, Germany
[2] Institute of Neuroimmunology and Multiple Sclerosis, ZMNH, University Medical Center Hamburg-Eppendorf, Falkenried 94 20251 Hamburg, Germany
[3] University of Hamburg, Division of Neurophysiology, 20146 Hamburg, Germany
[4] current address: Department of Psychological and Brain Sciences, Boston University, Boston, United States

Corresponding author:
* Christine Gee
Institute of Synaptic Physiology, ZMNH
University Medical Center Hamburg-Eppendorf
Falkenried 94
D-20251 Hamburg Germany
christine.gee@zmnh.uni-hamburg.de

Keywords: TRPM4, EAE, calcium imaging, electrophysiology, hippocampus

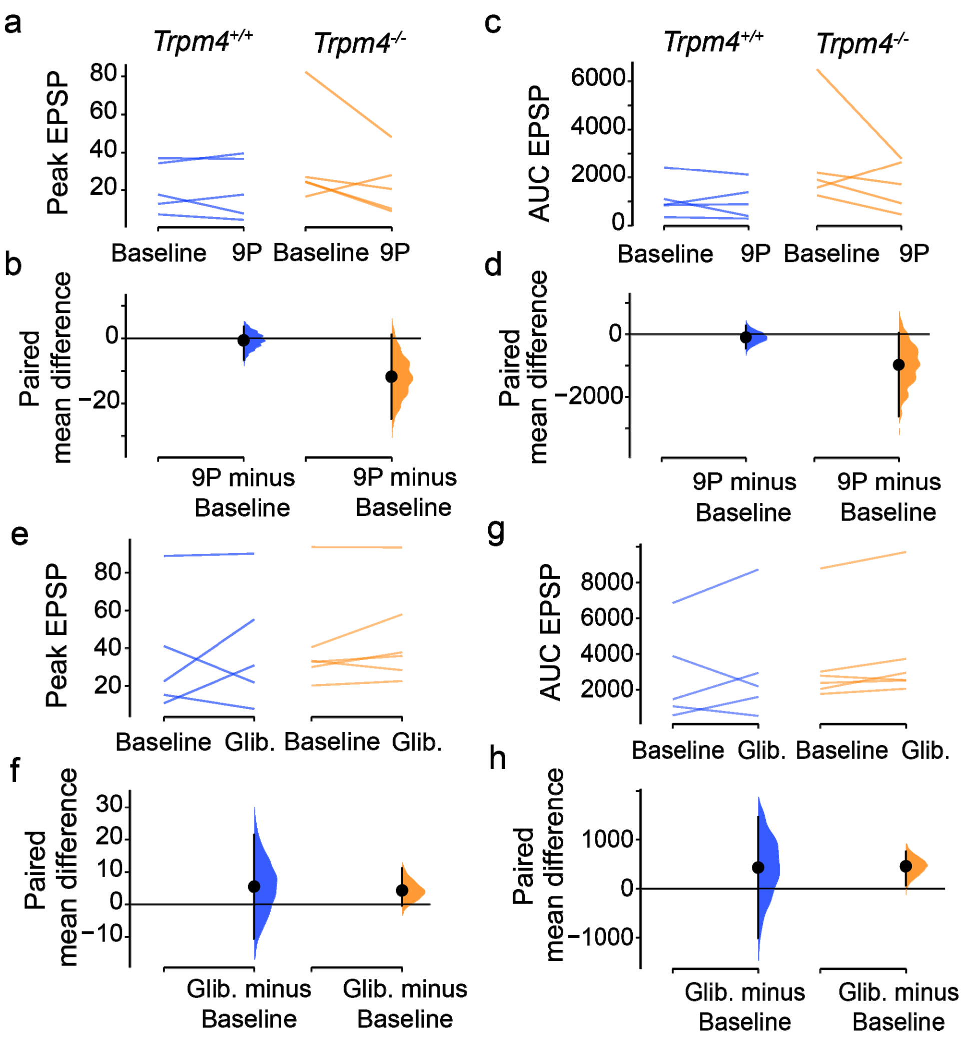


**Supplemental Figure 1: TRPM4 does not contribute to synaptic responses.** (a-d) Effect of 9-phenanthrol (9-P, 30 μM) on EPSPs recorded from CA1 pyramidal neurons in acute slices from wildtype (*Trpm4*$^{+/+}$) or knockout (*Trpm4*$^{-/-}$) littermates. (a, c) Raw peak or AUC EPSPs. (b, d) The paired mean differences are shown in Cumming estimation plots. (b) The paired mean difference between wildtype peak baseline and 9P is -0.609, p=0.805 [95.0%CI -6.63, 3.57]. The paired mean difference between knockout peak baseline and 9P is -11.8, p=0.196 [95.0%CI -24.7, 1.13]. (d) The paired mean difference between wildtype AUC baseline and 9P is -1.01e+02, p=0.619 [95.0%CI-4.49e+02, 2.66e+02]. The paired mean difference between knockout AUC baseline and 9P is -9.78e+02, p=0.245 [95.0%CI 2.61e+03,38.9]. (e-h) Effect of glibenclamide (Glib. 20 μM) on EPSPs recorded from CA1 pyramidal neurons in slices from wildtype (*Trpm4*$^{+/+}$) or knockout (*Trpm4*$^{-/-}$) littermates. (e, g) Raw peak or AUC EPSPs. (f, h) The paired mean differences are shown in Cumming estimation plots. (f) The paired mean difference between wildtype peak baseline and Glib. is 5.49, p=0.508 [95.0%CI -10.5, 21.5]. The paired mean difference between knockout peak baseline and Glib. is 4.3, p=0.268 [95.0%CI -0.352, 11.1]. (h) The paired mean difference between wildtype AUC Baseline and Glib. is 4.3e+02, p=0.498 [95.0%CI -1e+03,1.46e+03]. The paired mean difference between knockout AUC baseline and Glib. is 4.56e+02, p=0.0956 [95.0%CI 66.0,7.55e+02]. Mean differences are depicted as dots; 95% confidence intervals are indicated by the ends of the vertical error bars. Each paired mean difference is plotted as a bootstrap sampling distribution. A two-sided permutation t-test was used.

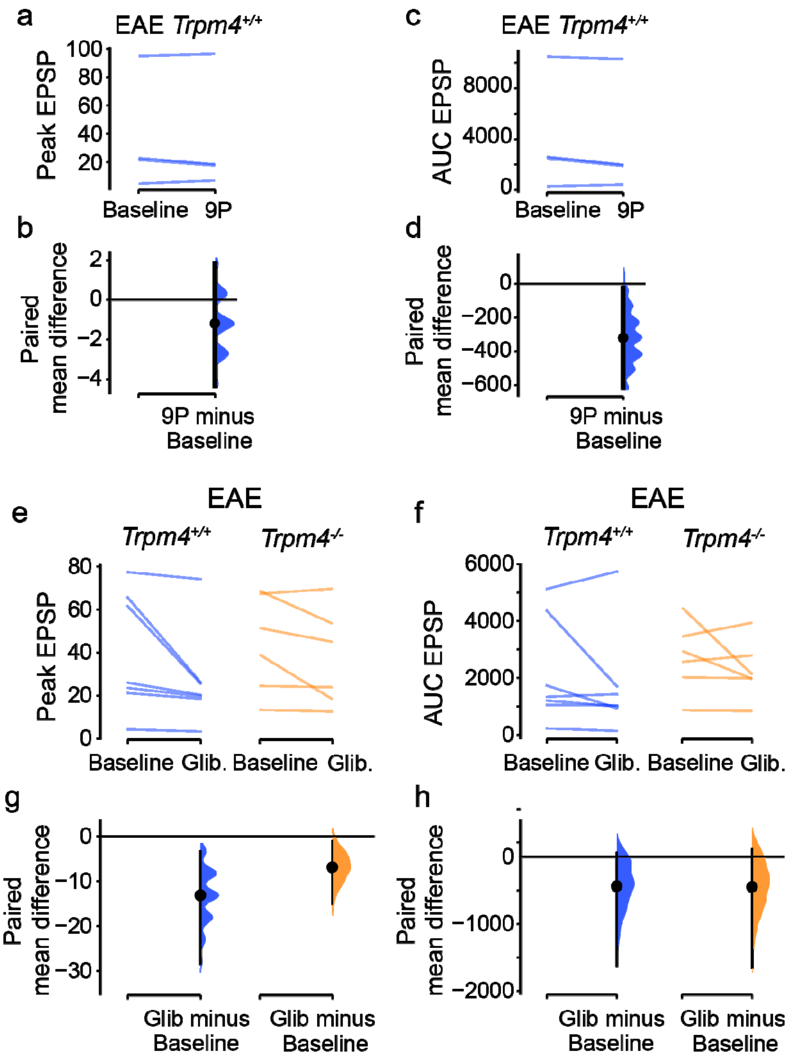


**Supplemental Figure 2: Glibenclamide, not 9-phenanthrol, reduces EPSP peak amplitude in CA1 pyramidal neurons in EAE mice** (a-d) Effect of 9-phenanthrol (9-P, 30 µM) on EPSPs recorded from wildtype (*Trpm4*$^{+/+}$) CA1 pyramidal neurons in acute slices prepared during the acute response to EAE. (a, c) Raw peak or AUC EPSPs. (b, d) The paired mean differences are shown in Cumming estimation plots. (b) The paired mean difference between wildtype peak baseline and 9P is -1.19, p=0.511 [95.0%CI -4.31, 1.83]. (d) The paired mean difference between wildtype AUC baseline and 9P is -3.21e+02, p=0.262 [95.0%CI-6.15e+02, -26.6]. (e-h) Effect of glibenclamide (Glib. 20 µM) on EPSPs recorded from CA1 pyramidal neurons from wildtype (*Trpm4*$^{+/+}$) or knockout (*Trpm4*$^{-/-}$) littermates in slices prepared during the acute response to EAE. (E, G) Raw peak or AUC EPSPs. (f, h) The paired mean differences are shown in Cumming estimation plots. (f) The paired mean difference between wildtype peak baseline and Glib. is -13.1, p=0.122* [95.0%CI -28.4, -3.4]. The paired mean difference between knockout peak baseline and Glib. is -6.91, p=0.158 [95.0%CI -15.1, -1.11]. (h) The paired mean difference between wildtype AUC Baseline and Glib. is -4.37e+02, p=0.306 [95.0%CI-1.63e+03, 60.5]. The paired mean difference between knockout AUC baseline and Glib. is -4.48e+02, p=0.382 [95.0%CI -1.65e+03,1.12e+02]. Mean differences are depicted as dots; 95% confidence intervals are indicated by the ends of the vertical error bars. Each paired mean difference is plotted as a bootstrap sampling distribution. A two-sided permutation t-test was used.

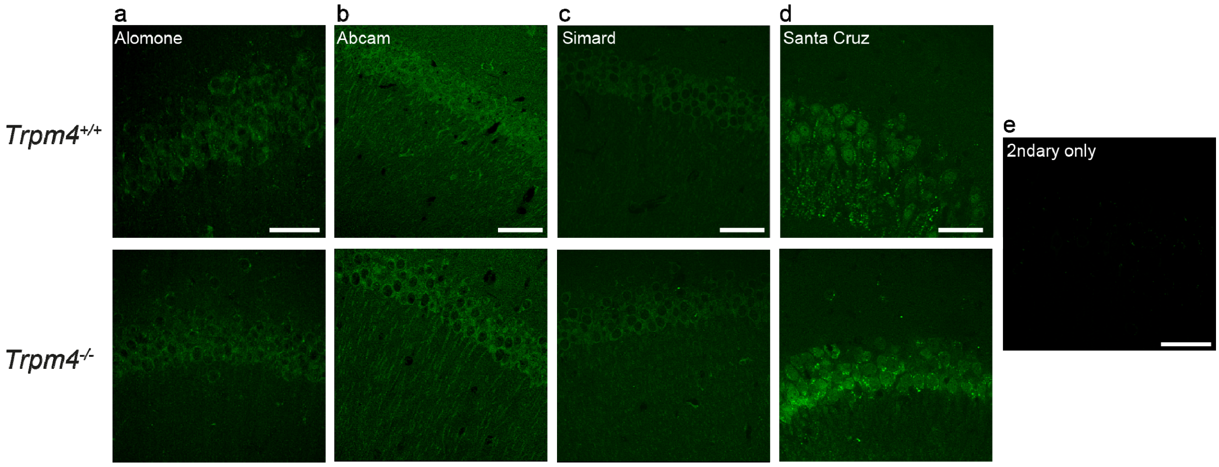


**Supplemental Figure 3: Indistinguishable anti-TRPM4 immunostaining in CA1 of *Trpm4*$^{-/-}$ and *Trpm4*$^{+/+}$ hippocampus.** (a-d) Confocal images (single optical sections) of the CA1 region of hippocampal sections immunostained using three rabbit anti-TRPM4 primary antibodies (a-c) and one goat anti-TRPM4 antibody (d). Top images are sections from *Trpm4*$^{+/+}$ and lower images from *Trpm4*$^{-/-}$ mice. (e) Confocal image from a section treated identically as those in A-C but without the primary antibody. The imaging conditions and applied lookup tables were identical for all images. Note the lack of differential staining in slices from mice with and without TRPM4 Scale bars are 50 µm.
